# Geographic distribution and predictors of diagnostic delays among possible TB patients in Uganda

**DOI:** 10.5588/pha.23.0010

**Published:** 2023-09-21

**Authors:** E. Ochom, K. O. Robsky, A. J. Gupta, A. Tamale, J. Kungu, P. Turimumahoro, S. Nakasendwa, I. B. Rwego, W. Muttamba, M. Joloba, W. Ssengooba, J. L. Davis, A. Katamba

**Affiliations:** 1 Uganda Tuberculosis Implementation Research Consortium, Kampala, Uganda;; 2 Department of Epidemiology, Johns Hopkins Bloomberg School of Public Health, Baltimore, MD,; 3 Department of Epidemiology of Microbial Diseases, Yale School of Public Health, New Haven, CT,; 4 Johns Hopkins Bloomberg School of Public Health, Baltimore, MD, USA; 5 Departments of Veterinary Medicine and Animal Resources,; 6 Biotechnical and Biolab Sciences, and; 7 Biosecurity, Ecosystem and Veterinary Public Health, College of Veterinary Medicine, Animal Resources and Biosecurity, Makerere University, Kampala, Uganda,; 8 Lung Institute, and; 9 Department of Medical Microbiology, College of Health Sciences, Makerere University, Kampala, Uganda;; 10 Pulmonary, Critical Care and Sleep Medicine Section, Yale School of Medicine, New Haven, CT, USA;; 11 Clinical Epidemiology Unit, Makerere University, College of Health Sciences, Kampala, Uganda

**Keywords:** tuberculosis, delayed diagnosis, GIS, Africa

## Abstract

**BACKGROUND:**

Understanding the geographic distribution and factors associated with delayed TB diagnosis may help target interventions to reduce delays and improve patient outcomes.

**METHODS:**

We conducted a secondary analysis of adults undergoing TB evaluation within a public health demonstration project in Uganda. Using Global Moran’s I (GMI) and Getis-Ord GI* statistics, we evaluated for residential clustering and hotspots associated with patient-related and health system-related delays. We performed multivariate logistic regression to identify individual predictors of both types of delays.

**RESULTS:**

Of 996 adults undergoing TB evaluation (median age: 37 years, IQR 28–49), 333 (33%) experienced patient delays, and 568 (57%) experienced health system delays. Participants were clustered (GMI 0.47–0.64, *P* ⩽ 0.001) at the sub-county level, but there were no statistically significant hotspots for patient or health system delays. Married individuals were less likely to experience patient delays (OR 0.6, 95% CI 0.48–0.75; *P* < 0.001). Those aged 38–57 years (OR 1.2, 95% CI 1.07–1.38; *P* = 0.002) were more likely than those aged ⩾58 years to experience patient delays. Knowledge about TB (OR 0.8, 95% CI 0.63–0.98; *P* = 0.03) protected against health system delays.

**CONCLUSIONS:**

We did not identify geographic hotspots for TB diagnostic delays. Instead, delays were associated with individual factors such as age, marital status and TB knowledge.

## Abstract (fr)

**CONTEXTE:**

Comprendre la distribution géographique et les facteurs associés aux retards de diagnostic de la TB peut aider à cibler les interventions visant à réduire les retards et à améliorer les résultats pour les patients.

**MÉTHODES:**

Nous avons effectué une analyse secondaire des adultes soumis à une évaluation de la TB dans le cadre d’un projet de démonstration de santé publique en Ouganda. À l'aide des statistiques Global Moran’s *I* (GMI) et Getis-Ord GI*, nous avons évalué les regroupements résidentiels et les points critiques associés aux retards liés aux patients et au système de santé. Nous avons effectué une régression logistique multivariée pour identifier les prédicteurs individuels des deux types de retards.

**RÉSULTATS:**

Sur les 996 adultes soumis à une évaluation de la TB (âge médian : 37 ans, IQR 28–49), 333 (33%) ont subi des retards liés aux patients et 568 (57%) ont subi des retards liés au système de santé. Les participants étaient regroupés (GMI 0,47–0,64 ; *P* ⩽ 0,001) au niveau du sous-comté, mais il n’y avait pas de points critiques statistiquement significatifs pour les retards des patients ou du système de santé. Les personnes mariées étaient moins susceptibles de subir des retards de la part des patients (OR 0,6 ; 95% CI 0,48–0,75 ; *P* < 0,001). Les personnes âgées de 38 à 57 ans (OR 1,2 ; 95% CI 1,07–1,38 **;*** P* = 0,002) étaient plus susceptibles que celles âgées de ⩾58 ans de subir des retards. Les connaissances sur la TB (OR 0,8 ; 95% CI 0,63–0,98 **; ***P* = 0,03) protégeaient contre les retards du système de santé.

**CONCLUSIONS:**

Nous n’avons pas identifié de points critiques géographiques pour les retards de diagnostic de la TB. Les retards étaient plutôt associés à des facteurs individuels tels que l’âge, la situation matrimoniale et les connaissances sur la TB.

TB is among the leading causes of morbidity and mortality worldwide,^1^ and patient-related delays in seeking healthcare and healthcare-related delays in diagnostic evaluation may be major contributors.^2^ Globally, a wide variety of individual and health-system characteristics have been associated with these delays, including female sex, low educational attainment, and receiving care from private providers, including traditional healers.^3^–^5^

Patient-related delays in seeking care and health-system delays in diagnosis and treatment initiation can both have severe consequences for the patient,^6^ and are unfortunately widespread. In low- and middle-income countries, up to 42% of patients delay seeking care when experiencing TB symptoms^5^ and 47–63% experience health system delays in diagnosis.^7^–^9^ In Uganda, patient and health system delays are 50%^10^ and 80%, respectively.^11^ Patients who develop TB symptoms and delay seeking healthcare risk developing more advanced forms of TB, which may increase mortality.^12^ Patients experiencing delays in TB diagnosis may also contribute to ongoing transmission to susceptible community members and perpetuate the spread of resistant strains.^12^,^13^

Reducing patient delays to seeking healthcare and health system delays may be critical to reducing TB morbidity and mortality. One possible solution is targeting public health interventions toward those most likely to experience these delays. A large and growing literature has used geographic analyses to understand the distribution of TB case notifications.^14^ However, more information about hotspots for patients experiencing diagnostic delays is necessary. Such data could help inform the design of more accessible and affordable TB evaluation and treatment. Therefore, we sought to determine the geographic distribution of possible TB patient and health-system delays in Uganda and identify demographic, socio-economic and clinical factors associated with these outcomes.

## METHODS

### Study design and population

We conducted a cross-sectional, secondary analysis using data from the East African Public Health Laboratory Network (EAPHLN) study at four Ugandan regional referral hospitals. The four referral hospitals serve four different regional territories of Uganda: Mbale Hospital in the East, Arua Hospital in the North West, Gulu Hospital in the North and Mbarara Hospital in the South West. The primary aim of the parent study was to evaluate Xpert® MTB/RIF (Cepheid, Sunnyvale, CA, USA) performance and its role in clinical diagnostic algorithms in routine settings.^15^ The EAPHLN study consecutively enrolled adults (⩾18 years) with possible active TB (defined as having ⩾1 TB symptom, including cough of any duration, fever, chills, or night sweats) presenting to any of the four participating hospitals for evaluation of TB symptoms from October 2015 to August 2016. For the current study, we included the subset of possible TB patients who reported cough and submitted a sputum sample for Xpert testing or fluorescence (smear) microscopy (FM). We excluded patients missing microbiological test results or a known residential address.

### Study procedures of the EAPHLN study

Trained research assistants enrolled and interviewed consenting patients and captured demographic and clinical characteristics and TB knowledge using a standardised case record form. Patients then provided a sputum sample for Xpert testing and FM examination, and a blood sample for HIV testing. All data were double-entered into an electronic database (EpiData v2.0/2007; EpiData Association, Odense, Denmark), and we resolved any differences by review of the source data.

### Outcome definitions

We had two outcomes of interest: patient delays and health system delays. We defined patient-related delay as present if the time from cough onset until the first consultation at a formal healthcare facility (government, private or non-profit) was ⩾21 days. We excluded other locations, such as drug shops, pharmacies and herbalists, because these sites typically do not offer microbiologic testing. We defined health system delay as present if the time from the first formal healthcare consultation until receiving microbiologic TB test results was ⩾15 days.

### Data analysis

We compared patient demographic and clinical characteristics among those who experienced patient or health system delays and those who did not and described their geographic distribution at the sub-county level. We identified sub-county boundaries and regional referral hospital locations using data shape files from the Uganda Bureau of Statistics (UBOS)^16^ for sub-county administrative and health facilities in 2020. We validated patient-reported sub-counties of residence against the official UBOS list. In case of inconsistencies, we corrected for sub-county using the patient’s village and parish in the online land conflict mapping tool.^17^ We first created district and sub-county layers of maps served by each referral hospital. We then mapped patient residences to determine the number and proportions of participants with patient and health-system delays at the sub-county level. We assessed for clustering (global spatial autocorrelation) and hotspots (areas with higher than expected concentrations) of possible TB patients, patient delays and health-system delays using Global Moran’s Index (GMI) and Getis-Ord GI*. We used ArcGIS v10.7 (Environmental Systems Research Institute, Redlands, CA, USA) for mapping and spatial data management.

We performed bivariate analyses using mixed logistic regression models to assess the association of ­demographic and clinical characteristics and TB knowledge with patient and health-system delays using odds ratios (ORs) and 95% confidence intervals (CIs). We defined a patient to have basic TB knowledge if he or she correctly identified cough as a means of transmitting TB and affirmed that TB is curable. The mixed-effects logistic regression model allowed for estimation of associations between participant’s demographic, clinical characteristics and TB knowledge with outcome variables (patient and health-system delays), while accounting for potential correlation between participants within regional referral hospital level. We incorporated all variables with a significance level of *P* < 0.2 from the bivariate analysis into the multivariate regression models. We evaluated statistical significance based on a reference threshold of *P* < 0.05. We used Stata v14 (Stata Corp, College Station, TX, USA) for all regression analyses.

### Human subject’s protection

The Makerere University School of Public Health Higher Degrees Research and Ethics Committee, Kampala, and the Uganda National Council of Science of Technology (UNCST), Kampala, Uganda, approved this study.

## RESULTS

### Patient characteristics

**FIGURE 1:**
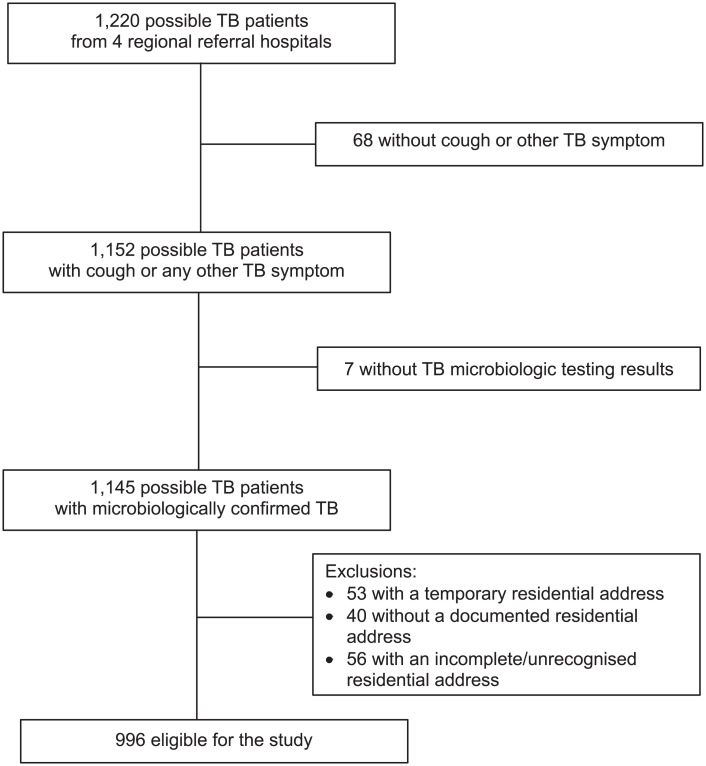
Flow diagram showing study inclusion and exclusion criteria. Microbiological confirmation of TB defined as one or more positive results on the Xpert^®^ MTB/RIF assay or fluorescence smear microscopy.

The EAPHLN study enrolled 1,220 adult possible TB patients from May 2015 to May 2016, including 996 eligible for the present sub-study (Figure 1). We excluded 68 who did not report cough or any other TB symptom, seven missing microbiological test results for both Xpert testing and FM microscopy, and 149 missing a permanent residential address. The study enrolled 181 (18%) patients from Mbale Referral Hospital, 389 (39%) from Arua Referral Hospital, 219 (22%) from Mbarara Referral Hospital and 207 (21%) from Gulu Referral Hospital. More than half of the participants (*n* = 567, 57%) were male. Most were 18–37 years of age (*n* = 508, 51%), and the majority were married (*n* = 645, 65%). In total, 34% (*n* = 337) reported living within 5 km of the referral hospital; 47% (*n* = 472) of patients had completed primary education; 78% (*n* = 781) were employed, but 81% (*n* = 635) of those employed reported earning less than USD5 per day; 13% (*n* = 131) had past TB disease. More than half of patients demonstrated basic knowledge of TB (*n* = 576, 58%). The prevalence of HIV in the study population was 28% (*n* = 281). Table 1 shows patient characteristics with patient delays, and Table 2 shows the characteristics of those with health-system delays.

**TABLE 1:** Characteristics associated with patient delays (*n* = 996)

Characteristics	Patient delays (*n* = 333, 33%) *n* (%)	No patient delays (*n* = 663, 67%) *n* (%)	Bivariate analysis		Multivariate analysis	
			OR (95% CI)	*P-*value	OR (95% CI)	*P-*value
Male sex	180 (54)	387 (58)	0.8 (0.72–0.97)	0.02	0.8 (0.73–1.01)	0.07
Age group, years						
18–37	163 (49)	345 (52)	1		1	
38–57	116 (35)	235 (35)	1.0 (0.98–1.11)	0.17	1.2 (0.07–1.38)	0.002
⩾58	54 (16)	83 (12)	1.2 (0.83–2.26)	0.21	1.4 (0.88–2.31)	0.15
Marital status: married	191 (57)	454 (68)	0.6 (0.50–0.76)	<0.001	0.6 (0.48–0.75)	<0.001
Travel distance to hospital, km*						
⩽5	115 (34)	222 (33)	1	—		
6–10	53 (16)	104 (16)	1.0 (0.44–2.19)	0.97	—	—
⩾11	165 (50)	337 (51)	0.9 (0.72–1.23)	0.68	—	—
Education						
No formal education	52 (16)	87 (13)	1	—		
Primary	172 (52)	300 (45)	0.9 (0.42–2.19)	0.92	—	—
Secondary	87 (26)	213 (32)	0.7 (0.28–1.63)	0.39	—	—
Tertiary	22 (07)	63 (10)	0.6 (0.17–2.03)	0.40	—	—
Employment status: employed Earned daily income,^†^ USD	251 (76)	530 (80)	0.8 (0.53–1.15)	0.20	—	—
<1	83 (33)	151 (28)	1	—		
1–5	121 (48)	280 (53)	0.8 (0.52–1.19)	0.25	—	—
>5	47 (19)	99 (19)	0.9 (0.39–1.91)	0.71	—	—
History of TB: yes	55 (17)	76 (11)	1.5 (0.77–3.02)	0.22	—	—
Had knowledge of TB: yes	189 (57)	387 (58)	0.9 (0.66–1.83)	0.74	—	—
Living with HIV: yes	85 (26)	196 (30)	0.8 (0.45–1.47)	0.50	—	—

* Participant travel distance from their residence to regional referral hospital.

† Among those employed only.

OR = odds ratio; CI = confidence interval; USD = United States dollar.

**TABLE 2:** Characteristics associated with health system delays (*n* = 996)

Characteristics	Health system delays (*n* = 568, 57) *n* (%)	No health system delays (*n* = 428, 43) *n* (%)	Bivariate analysis		Multivariate analysis	
			OR (95% CI)	*P-*value	OR (95% CI)	*P-*value
Male sex	329 (58)	238 (55)	1.1 (0.97–1.37)	0.09	1.1 (0.89–1.41)	0.33
Age group, years						
18–37	288 (51)	220 (51)	1			
38–57	200 (35)	151 (35)	1.0 (0.83–1.19)	0.99	—	—
⩾58	76 (13)	61 (14)	0.9 (0.64–1.35)	0.70	—	—
Marital status: married	369 (65)	276 (64)	1.1 (0.63–1.82)	0.78	—	—
Travel distance to hospital, km*						
⩽5	187 (35)	150 (33)	1			
6–10	78 (14)	79 (18)	0.8 (0.33–1.94)	0.62	—	—
⩾11	203 (47)	299 (53)	1.2 (0.58–2.65)	0.57	—	—
Education						
No formal education	68 (16)	71 (12)	1		1	
Primary	261 (46)	211 (49)	1.3 (1.11–1.61)	0.002	1.2 (0.89–1.72)	0.20
Secondary	186 (33)	114 (26)	1.7 (0.70–4.25)	0.23	1.7 (0.65–4.32)	0.28
Tertiary	49 (07)	36 (08)	1.3 (0.54–3.38)	0.51	1.4 (0.49–3.96)	0.53
Employment status: employed Earned daily income,^†^ USD	445 (79)	336 (78)	1.0 (0.59–1.87)	0.86	—	—
<1	131 (29)	103 (31)	1			
1–5	238 (53)	163 (48)	1.1 (0.83–1.60)	0.40	—	—
>5	76 (17)	70 (21)	0.8 (0.62–1.12)	0.25	—	—
History of TB: yes	79 (14)	52 (12)	1.2 (0.43–3.64)	0.67	—	—
Had knowledge of TB: yes	320 (57)	257 (59)	0.9 (0.71–1.07)	0.19	0.8 (0.63–0.98)	0.03
Living with HIV: yes	177 (31)	104 (24)	1.2 (0.67–2.11)	0.14	1.5 (0.88–2.71)	0.13

* Participant travel distance from their residence to regional referral hospital.

† Among those employed only.

OR = odds ratio; CI = confidence interval; USD = United States dollar.

### Distribution of patients

The geographic distribution of patients seeking TB services at the referral hospitals was heterogeneous, with not all sub-counties in the catchment districts reporting individuals. Of 239 sub-counties within 17 Mbale Referral Hospital catchment districts, 44 sub-counties registered patients. Arua had 37 of 98 sub-counties with possible TB patients in 8 districts. Mbarara had 67 of 254 sub-counties with possible TB patients in 20 districts, and Gulu had 66 of 234 sub-counties with possible TB patients in 25 districts.

Patient delays to seeking healthcare were 32–37% (Arua: *n* = 125, 32%; Gulu: *n* = 66, 32%; Mbale: *n* = 60, 33%; Mbarara: *n* = 82, 37%) across all health facilities. Health-system delays were more common than patient delays across all referral hospitals; 39–70% of individuals experienced health-system delays (Gulu: *n* = 80, 39%; Arua: *n* = 216, 56%; Mbale: *n* = 118, 65%; Mbarara: *n* = 154, 70%). Some patients experienced both patient and health-system delays (Mbale: *n* = 1, 0.5%; Arua: *n* = 1, 0.2%; Mbarara: *n* = 57, 26%; Gulu: *n* = 22, 11%). The number of patients who did not experience any of the delays were as follows: 4 (2%) in Mbale, 49 (12%) in Arua, 40 (18%) in Mbarara and 83 (40%) in Gulu.

**FIGURE 2:**
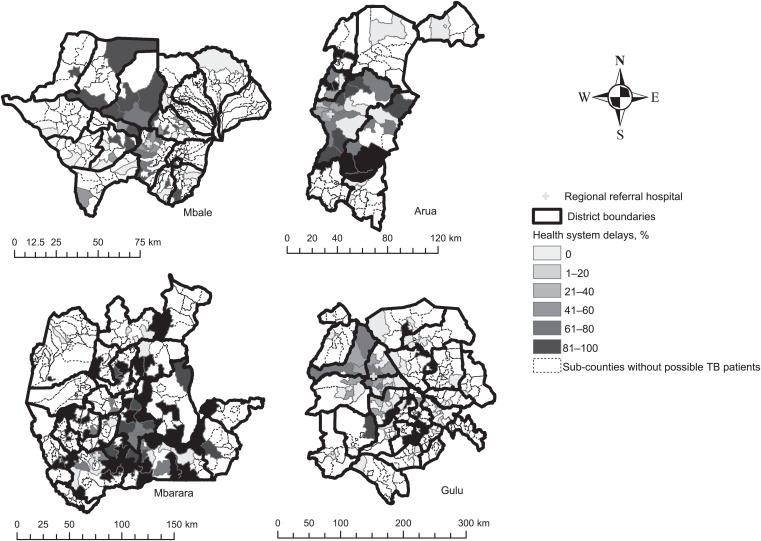
Distribution of possible TB patients experiencing health-system delays. The GMI for health-system delays was –0.12 for Mbale (*P* = 0.58), 0.13 for Arua (*P* = 0.13), –0.03 for Mbarara (*P* = 0.86) and –0.07 for Gulu (*P* = 0.61).

There was a clustering of possible TB patients at the sub-county level in each of the four referral hospital catchment areas. GMI values were 0.47–0.64 with *P* ⩽ 0.001. In contrast, we did not observe clustering of patient or health-system delays in the primary catchment areas in any of the four referral hospitals. We did not observe any hotspots for possible TB patients or health-system delays using Getis-Ord GI* (Figure 2).

### Predictors of delays

There were 333 (33%) possible TB patients who experienced delays in formally seeking healthcare. The median duration of delay among patients was 32 days (interquartile range [IQR] 30–60). More than half (*n* = 568, 57%) experienced health-system delays. The median duration of health-system delay was 60 days (IQR 32–92). Just over 8% (*n* = 81) of possible TB patients experienced both patient and health-system delays, while almost a fifth (*n* = 176, 18%) of all possible TB patients did not experience any delay.

Being married (OR 0.6, 95% CI 0.48–0.75; *P* < 0.001*) *was associated with a decreased likelihood of patient delay in seeking healthcare*. *Patients aged 38–57 years were more likely to experience delays in seeking healthcare (OR 1.2, 95% CI 1.07–1.38; *P* = 0.002). No other factors, including self-reported distance from home to the facility, were associated with patient delays (Table 1). Knowledge of TB was associated with a decreased likelihood of experiencing health-system delays (OR 0.8, 95% CI 0.63–0.98; *P* = 0.03). No other factors, including self-reported distance from home to the facility, were associated with health-system delays (Table 2).

## DISCUSSION

We observed that about a third of possible TB patients were delayed in formally seeking healthcare, and over half experienced health-system delays. While we also observed clustering of possible TB patients at the sub-county level for all four referral hospitals, there was little evidence of clustering of patient and health-system delays. This finding suggests that location of residence does not play a significant role in delayed TB diagnosis. Our analysis also suggests that patient and health-system delays were comparable in frequency among those living near the referral hospitals in the study and those living further away. Being married was associated with seeking healthcare earlier than being unmarried. While beyond the scope of our study, previous studies have shown that partners of patients may directly or indirectly encourage earlier healthcare-seeking in the United States,^18^ Tanzania, Malawi and South Africa.^19^ The influence of partners may represent an opportunity for health systems to engage patients and their significant others in promoting timely visits to health centres. The study also observed that possible TB patients in the 38–57-year age group were likely to delay seeking formal healthcare. Our study findings were aligned with other studies in Mongolia^20^ and Ethiopia,^21^ while other studies reported patient delays in older age groups.^22^,^23^ In addition, knowledge of TB was protective against health-system delays. In this study, we were unable to explore these relationships fully. However, future studies should investigate the relationship between patient knowledge and the benefits of completing TB diagnosis early. Studies might also explore whether past experiences in these public health facilities influence the occurrence of patient delays.

Guidelines seeking to enhance TB screening/diagnostic services have not reduced delays, partly because of implementation challenges.^24^,^25^ We found that patient delays were less common than health-system delays. However, both kinds of delays were geographically widespread. We also showed that possible TB patients are clustered in certain areas, which could help direct case-finding efforts. Educating health providers is necessary to increase TB evaluation in all symptomatic patients in such areas.

Our study had limitations. First, excluding patients with extrapulmonary TB or pulmonary TB patients diagnosed using chest X-ray or based on clinical judgment may have reduced the mean duration of delays, especially for health-system delays.^20^ However, the absolute number of patients with an extrapulmonary or radiographic TB diagnosis is usually modest, making it unlikely that including these individuals would have substantially altered our findings. Second, aggregating individuals by sub-county may mask heterogeneity within smaller geographic units. However, this was necessary because our sample size did not allow us to look at clustering in smaller geographic areas. Moreover, any interventions to reduce delays in diagnosis are likely to be implemented at the sub-county level or higher. Third, because the number of observations within individual sub-counties was low, larger studies may be required to increase the power and accuracy of the findings within sub-counties. Fourth, a small proportion of observations were excluded from multivariate analysis due to missing geographic data. However, the sample size for multivariate analysis was adequate for analysis of factors of patient and health-system delays.

## CONCLUSION

Patient and health-system delays were not concentrated in geographic hotspots but widespread. Delays were instead associated with individual factors such as marital status, age and TB knowledge but not geographical location. Patient education and improving referral systems within clinical settings may help reduce delays in diagnosis among those with poor knowledge of TB.
